# Spatiotemporal Analysis of Multichannel EEG: CARTOOL

**DOI:** 10.1155/2011/813870

**Published:** 2011-01-05

**Authors:** Denis Brunet, Micah M. Murray, Christoph M. Michel

**Affiliations:** ^1^Functional Brain Mapping Laboratory, Departments of Fundamental and Clinical Neurosciences, University Medical School, University of Geneva, 1 rue Michel-Servet, 1211 Geneva, Switzerland; ^2^EEG Brain Mapping Core, Center for Biomedical Imaging (CIBM), 1211 Geneva, Switzerland; ^3^The Functional Electrical Neuroimaging Laboratory, Department of Clinical Neurosciences and Department of Radiology, Vaudois University Hospital Center, University of Lausanne, 1011 Lausanne, Switzerland

## Abstract

This paper describes methods to analyze the brain's electric fields recorded with multichannel Electroencephalogram (EEG) and demonstrates their implementation in the software CARTOOL. It focuses on the analysis of the spatial properties of these fields and on quantitative assessment of changes of field topographies across time, experimental conditions, or populations. Topographic analyses are advantageous because they are reference independents and thus render statistically unambiguous results. Neurophysiologically, differences in topography directly indicate changes in the configuration of the active neuronal sources in the brain. We describe global measures of field strength and field similarities, temporal segmentation based on topographic variations, topographic analysis in the frequency domain, topographic statistical analysis, and source imaging based on distributed inverse solutions. All analysis methods are implemented in a freely available academic software package called CARTOOL. Besides providing these analysis tools, CARTOOL is particularly designed to visualize the data and the analysis results using 3-dimensional display routines that allow rapid manipulation and animation of 3D images. CARTOOL therefore is a helpful tool for researchers as well as for clinicians to interpret multichannel EEG and evoked potentials in a global, comprehensive, and unambiguous way.

## 1. Introduction

**Figure 1:**
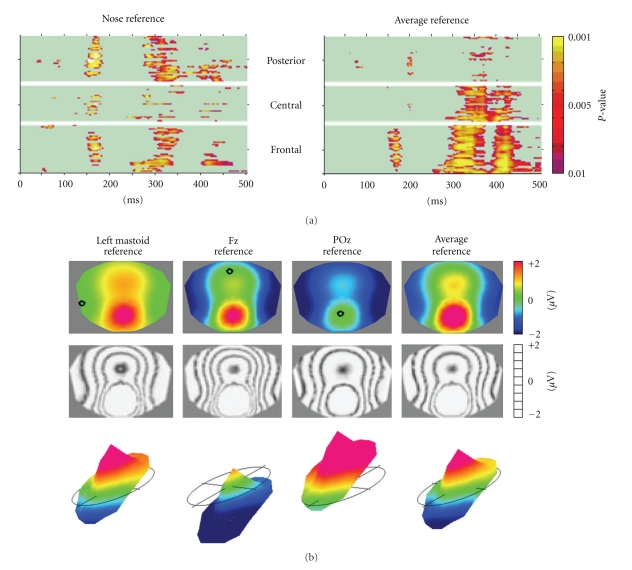
The reference issue in EEG: EEG waveform analysis is reference-dependent while topographical analysis is not. (a) Statistical comparison of evoked potential waveforms of two conditions (illusory contours versus no contours from [10]) for each electrode and each time point. Left: All electrodes referenced to the nose, right: all electrodes referenced to the Average Reference. Note the change of the results in time as well as in space. (b) Scalp potential map (seen from top) referenced to different electrodes. Top: color maps. Middle: same maps displayed with isopotential lines. Bottom: same maps displayed in relief with the zero level indicated. Note that the topography of the maps does not change, only the zero line and thus the color codes change.

The traditional analysis of the electroencephalogram (EEG) and event-related potentials (ERPs) focuses on waveform morphology over time at certain electrode positions. Scalp sites of interest are selected and the time course of the potential recorded at any site is analyzed using a variety of signal processing tools in the time and frequency domains. While this approach has provided many important insights into normal and pathological neuronal activity, it disregards another important dimension that multichannel EEG offers: the spatial characteristics of the electric fields at the scalp and the temporal dynamics of these fields. Any distribution and orientation of the active neurons at a given moment in time will generate a certain electric field on the scalp surface due to volume conduction [1]. While different generator configurations can lead to the same scalp fields, the inverse is not true: different scalp fields must have been generated by different configurations of generators in the brain [2, 3]. Consequently, analyzing the electric field topography, that is, the configuration of the potential isocontour maps on the scalp, and looking for topographical differences allows to detect moments when different neuronal populations were active in the brain, being it in time, between experimental conditions or under given pathological circumstances. Besides this direct neurophysiological interpretability, the analysis of the topography of the electric fields has another important advantage as compared to the analysis of waveforms: it is completely reference independent. The recording reference does not influence the topography of the scalp electric field and thus does not influence global topographic measures [4–6]. This is not true for methods that analyze the EEG or evoked potential waveforms. There is no point that records zero potential over time [4]. Consequently, changing the reference electrode changes the waveform shapes at each recording electrode. Therefore, any statistical comparison of amplitudes at a given electrode between conditions will change when the reference has changed, making the results ambiguous [7–9]. By contrast, the map topography does not change when changing the reference. Only the zero line is shifted, but not the landscape of the potential map [3]. Consequently, all analysis methods based on map topography are reference independent and unambiguous. This important argument for the spatial analysis of the EEG is illustrated in Figure 1.

 With the program CARTOOL, which exists now since over 14 years with constantly increasing capabilities, we wanted to provide an analysis tool for those researchers and clinicians who are interested in such reference-free EEG mapping techniques. It started with dynamic displays of EEG maps and calculation of some basic quantitative topographic measures. Soon after, standard data preprocessing tools were implemented, such as interpolation of electrodes, filtering, averaging, rereferencing. The next versions implemented spatial analysis methods for spontaneous and event-related EEG, most importantly the spatial microstate segmentation, initially proposed by Lehmann and collaborators [11], and subsequently advanced by cluster analysis and fitting methods. Also, other statistical topographic analysis methods were implemented. With the development of distributed inverse solutions and the advancement of computer power, source estimations in realistic head models were integrated. Important aspects of the software are the 3-dimensional visualization of the data as well as the fast display of the temporal dynamics of the scalp electric fields and the corresponding estimated sources. For that, the software puts particular emphasis on interactive manipulation and synchronization of the different windows by the user, using mouse and keyboard commands.

In the following we will describe some of the main methods for the spatial analysis of the scalp electric fields and how they are implemented in CARTOOL. Not all methods will be covered here, but they will give an impression of how multichannel EEG and ERP data can be analyzed in a comprehensive way. More details regarding the different spatial analysis methods can be found in the book “Electrical Neuroimaging” [12].

## 2. Data Preprocessing

**Figure 2:**
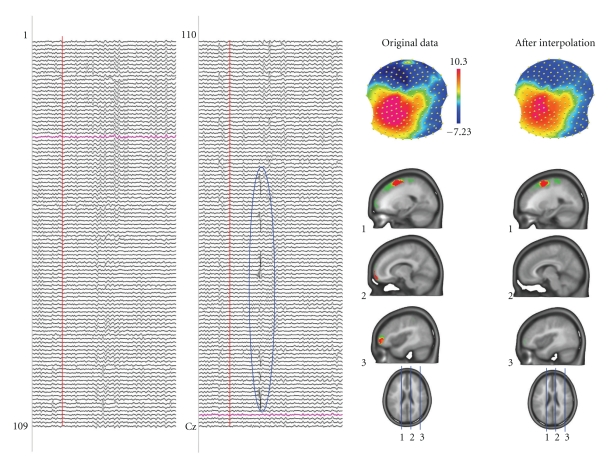
Artifact detection by inspecting the potential maps. The left panel shows spontaneous EEG recorded from 204 electrodes. Some artifacts, like the one encircled, are easy to see in the traces and such epochs can be eliminated. However, other artifacts are not easy to see in the traces but are readily detected in the maps by isolated “islands” of potential of a certain electrode. In this example a mid-frontal and a right frontal electrode are artifact contaminated. They generate steep gradients in the electric field and consequently produce strong sources in the inverse solution (here LAURA). Interpolating these electrodes using spline interpolation eliminates the bad electrodes and the sources caused by these artifacts disappear.

Topographic analysis of the electric field at the scalp very crucially depends on the quality of the data at each channel. Contamination by bad or noisy electrodes can lead to steep local gradients that have no neurophysiologic basis, which can in turn obfuscate interpretability of results (particularly those of source localization). Artifacts on one particular channel are not always easy to detect if only EEG waveforms are displayed. In contrast, contaminated channels are readily seen on time series of EEG maps because they behave differently from the neighboring channels and appear as isolated “spots” in the maps (Figure 2; see also [13]). 

**Figure 3:**
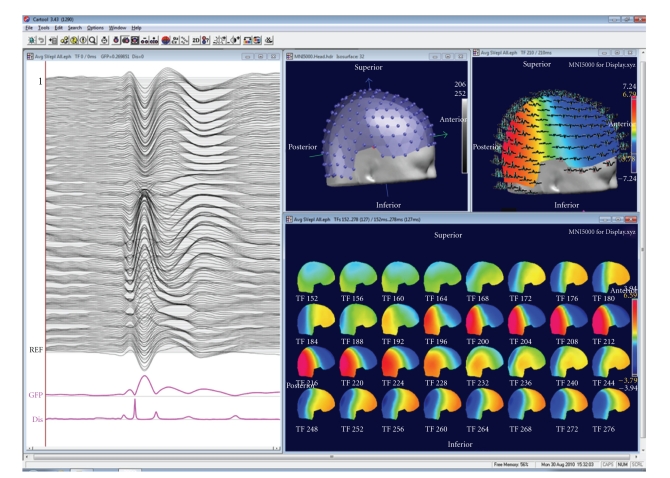
Example of a basic display window in CARTOOL. The individual tracks are displayed together with the Global Field Power and the Global Map Dissimilarity on the left. Tracks can also be displayed in 3D on the head surface as shown on the top right. Electrode positions and maps can be displayed in 3D or 2D. Maps can be shown at a single time point at cursor position, as animation over time or as time series of maps within a selected window.

CARTOOL provides various options to display EEG maps in 2D and 3D and with dynamic color scaling (Figure 3). Maps can be displayed in series over time or as animated movies. Electrode positions and electrode names can be displayed and marked both on the maps and on the waveforms. After clicking on bad channels, an interpolation tool is available that interpolates these channels using any of several different types of interpolation methods (surface spline, spherical spline or 3D spline) [14]. The 3D spline interpolation accounts for the real geometry of the head and is recommended if the real position of the electrodes is available. The interpolation toolbox also allows transforming the individual data of different subjects with different electrode positions to a common coordinate system for further statistical processing across subjects.

Once the data are cleaned from bad electrodes and interpolated, several standard preprocessing tools are available in CARTOOL, such as:

- filtering using 2nd-order Butterworth filters with −12 dB/octave roll-off,
- DC removal and Notch filter, envelope filter by rectifying absolute or squared values.
- downsampling by a Cascaded Integrator Comb filter followed by a high-pass FIR and decimation,
- recalculation against any type of single or combined electrode reference including current density (i.e., 2nd spatial derivative),
- exporting single or multiple tracks before or after applying the above preprocessing steps.

For evoked potential analysis, single or averaged epochs can be calculated for any combination of triggers or markers with or without baseline correction. Automatic artifact detection and epoch rejection using amplitude windows is available. CARTOOL puts a lot of emphasis in a flexible visualization of the EEG tracks during evoked potential analysis for manual determination of artifacts. During the evoked potential analysis, a trigger validation file is generated that can later be used to more rapidly redo the averaging with the same epochs.

In addition to the epoching according to defined triggers, CARTOOL allows to set markers according to specific characteristics in certain channels. This allows, for example, one to set markers at the onset of a motor response recorded with the EMG or at the peak of epileptic spikes. 

Finally, a file calculator tool has been implemented in CARTOOL that allows applying preset as well as user-defined mathematical operations to different files in a batch-mode processing.

## 3. Global Topographic Measures

In the default display mode, CARTOOL always shows two global topographic measures together with the waveforms and the maps (Figure 3). These two global measures are the Global Field Power and the Global Map Dissimilarity [5]. They are considered as additional measures and can be treated in the same way as the different tracks of the electrodes. The Global Field Power (GFP) is the standard deviation of the potentials at all electrodes of an average-reference map. It is defined as

(1) $$\text{GFP}\;=\sqrt{\frac{\sum_{i=1}^{N}{\left(u_{i}-\overset{̅}{u}\right)}^{2}}{N}},$$

where *u*_*i*_ is the voltage of the map *u* at the electrode *i*, $\overset{̅}{u}$ is the average voltage of all electrodes of the map *u* and *N* is the number of electrodes of the map *u*. Scalp potential fields with pronounced peaks and troughs and steep gradients will result in high GFP, while GFP is low in maps which have a “flat” appearance with shallow gradients. GFP is a one-number measure of the map at each moment in time. Displaying this measure over time allows to identify moments of high signal-to-noise ratio, corresponding to moments of high global neuronal synchronization [15, 16]. GFP can also be used to normalize data across subjects by dividing each map (i.e., the voltage at each electrode) by the mean GFP over time. General intra individual differences of the surface potential, for example due to difference in skull conductivity, can thereby be adjusted.

The Global Map Dissimilarity measure (GMD) is a measure of topographic differences of scalp potential maps. It is defined as

(2) $$\text{GMD}\;=\sqrt{\frac{1}{N}\overset{N}{\underset{i=1}{\sum }}{\left\{\frac{u_{i}-\overset{̅}{u}}{\sqrt{\sum_{i=1}^{N}{\left(u_{i}-\overset{̅}{u}\right)}^{2}/N}}-\frac{v_{i}-\overset{̅}{v}}{\sqrt{\sum_{i=1}^{N}{\left(v_{i}-\overset{̅}{v}\right)}^{2}/N}}\right\}}^{2}},$$

where *u*_*i*_ is the voltage of map *u* at the electrode *i*, *v*_*i*_ is the voltage of map *v* at the electrode *i*, $\overset{̅}{u}$ is the average voltage of all electrodes of map *u*, $\overset{̅}{v}$ is the average voltage of all electrodes of map *v*, and *N* is the total number of electrodes. In order to assure that only topography differences are taken into account, the two maps that are compared are first normalized by dividing the potential values at each electrode of a given map by its GFP. The GMD is 0 when two maps are equal, and maximally reaches 2 for the case where the two maps have the same topography with reversed polarity. Figure 2 in [17] illustrates the definition of the GMD. 

The GMD is equivalent to the spatial Pearson's product-moment correlation coefficient between the potentials of the two maps to compare [18]. The calculation of the GMD is a first step for defining whether different sources are involved in generating the electrical activity at the scalp for the two processes/populations being compared. If two maps differ in topography independently of their strength, it directly indicates that the two maps were generated by a different configuration of sources in the brain. As will be described later, all statistical topographic analysis methods in CARTOOL that compare topographies between conditions or groups use GMD (or the spatial correlation) as the basic measure of map similarity. GMD can also be used to compare topographies between successive time points. The display of the GMD across time then allows defining periods of map stability and moments of map changes. It is generally observed (particularly in evoked potentials) that GMD is inversely correlated with the GFP: GMD is high when GFP is low [19]. This observation indicates that maps tend to remain rather stable in topography during high GFP and change the configuration when GFP is low.

## 4. Microstate Segmentation

The display of the GMD across time has a very characteristic behavior which is similar for spontaneous EEG and for evoked potentials: the topography of the maps remains stable for several tens of milliseconds and then abruptly switches to a new configuration in which it remains stable again. This leads to periods of low GMD interrupted by sharp GMD peaks (Figure 3). This highly reproducible observation of periods of stable map topography has led to the concept of *functional microstates* first described by Lehmann et al. [11, 20]. The microstates correspond to a period of coherent synchronized activation of a large-scale neuronal network. Lehmann et al. proposed that the functional microstates represent the basic building blocks of information processing, the “atoms of thought”, being it spontaneous or evoked by a stimulus [21]. This corresponds well to the proposal that neurocognitive networks evolve through a sequence of quasistable coordination states, rather than a continuous flow of neuronal activity [22–25]. With respect to the ERPs, each successive microstate represents a certain information processing step that leads from perception to action [26]. While several parallel activations are possible and are most likely occurring in each step, there nonetheless seems to be a certain sequence of information processing, probably related to the integration of the information at different complexity levels [27].

In light of this interpretation of the observed sequential periods of map stability, different methods have been proposed to objectively and automatically define the different microstates and to statistically evaluate the specificity of certain microstates under given experimental conditions. CARTOOL has implemented these methods in the “microstate segmentation” and “map fitting” modules. The microstate segmentation is based on cluster analysis using either a modified k-means cluster analysis [28] or an atomize and agglomerate hierarchical cluster analysis with or without GFP normalization [17], followed by some temporal postprocessing steps. The k-means cluster analysis is a classical pattern recognition method used in many applications in different fields. It is an iterative procedure, starting with an initial guess of maps and terminating when successive iterations differ negligibly. Because of these iterations the result of the k-means cluster analysis can slightly vary from one run to the other. In contrast, the hierarchical cluster analysis that we devised specifically for microstate segmentation does not iterate and thus gives unique results. It is a modified agglomerative hierarchical clustering in a way that clusters that greatly contribute to the global explained variance are retained even if they are present for a short period of time only. More detailed explanation of the two methods can be found in [9].

**Figure 4:**
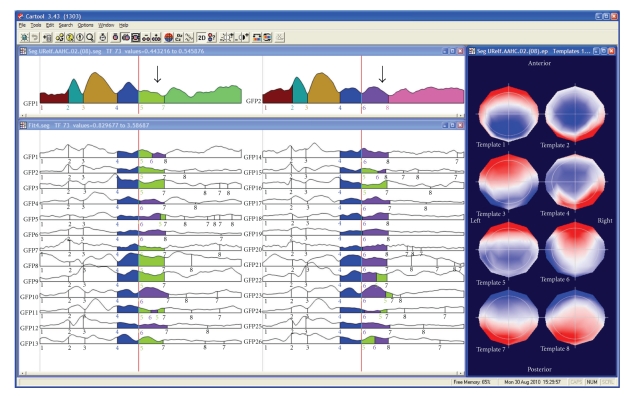
Illustration of the microstate segmentation in CARTOOL. The two windows on the top show the segments resulting from the k-means cluster analysis of the grand-mean ERP of two conditions. The segments are marked under the Global Field Power curves. Different colors indicate different segments. The cluster maps of these segments are displayed on the right. Note that in the beginning the same segments are found for the two conditions, while different segments explain the later components. Fitting the cluster maps to each single subject ERP statistically tests this finding. This is illustrated here by showing that more subjects have map number 5 (green) in condition 1 and map number 6 (purple) in condition 2. Duration, explained variance and other parameters are computed for each segment and can then be statistically compared using CARTOOL or any other statistical package.

The cluster analysis can be applied to one data file or to different files of different experimental conditions and/or populations (Figure 4). The result is a certain number of prototype maps (also called cluster maps) that best represent the whole data set. For defining the optimal number of cluster maps, CARTOOL proposes two criteria: a cross-validation criterion and the Krzanovski-Lai criterion [17]. The cross-validation is derived by dividing the global explained variance by the degrees of freedom, the latter depending on the number of electrodes. The Krzanovski-Lai criterion is determined by the L-corner of the dispersion curve, which is a quality measure of the clustering, meaning the optimal clustering is set when an additional cluster does not lead to a significant gain of the global quality (for details see [9]).

The cluster maps are finally fitted back to the original data and each time point is labeled with the cluster maps it correlated best with (in terms of GMD). In order to ensure a certain degree of continuity in time of the different a final relabeling step is performed which satisfies two requirements: (1) the correlation between the measurement and the cluster map should be high, and (2) the majority of the neighboring measurements should belong to the same microstate. Standard smoothing techniques, well-known in statistics, are used to fulfill this compromise between goodness of fit and smoothness [28]. CARTOOL allows adjusting these smoothness parameters. In addition, small segments can optionally be rejected. The result of the microstate segmentation is displayed color-coded under the GFP curve with each color representing a different cluster map. Additional options are available to sequentialize clusters and to merge highly correlated clusters. 

Another method that has been proposed to define the most dominant evoked component topographies in a dataset is based on an independent component analysis (ICA, [29]). It has been shown that both ICA and cluster analysis lead to rather similar results and thus have compatible underlying assumptions [30, 31]. However, the main limitation of ICA is that it assumes that global brain activity is generated by a superimposition of a number of independent processes. While this assumption might be valid in the case of artifacts such as eye movements or cardiac activity, it is difficult to accept for brain activity, where the principal organization relies on distributed neural networks with tightly linked cross-talk between the different areas. In such systems, the different components are dynamically coupled and cannot be separated in independent components. ICA would fail to uncover such processes, while the cluster analysis does not require such independence.

The cluster analysis of ERPs is usually applied to the group-averaged files. All experimental conditions/populations are entered into the cluster analysis, and the optimal number of clusters for the whole data set is determined [27, 32]. The cluster maps are then fitted to the data by calculating the spatial correlation (the GMD, see above) between each cluster map and each time point of the data. Each time point is then labeled with the cluster map with which it correlates best. It is interesting to note that this fitting procedure results in stable periods that are represented by the same cluster map even though no temporal constraint is a priori imposed, thus directly and empirically confirming the microstate model. The labeling procedure and the display of the results of this labeling as color-coded segments under the GFP curves allows the experimenter to generate hypotheses about the specificity of certain microstate maps for certain experimental conditions/populations (Figure 4).

It is important to emphasize that the microstate segmentation on the grand mean data allows hypothesis generation and is not the final result. Changing the number of clusters might change the results at this level by proposing more or less map differences across time or between conditions. A second statistical step is needed to confirm these hypotheses and define those microstates that remain statistically significant. The “microstate fitting” module of CARTOOL allows to perform this test. The fitting procedure is the same as for the grand mean, but now the cluster maps are fitted to the individual ERPs of each subject and each condition/population [32] (Figure 4). Several different parameters are then computed that describe the goodness of fit, the number of maps that each cluster explained, the onset and the offset of each cluster map, and so forth [17]. Spreadsheets are generated with these values that can directly be read into any statistical software package as well as into Excel, but that can also directly be used in the statistical analysis module in CARTOOL. Only microstates that are significantly different after this statistical fitting procedure are considered as stable. Users will realize that in most cases increasing the number of clusters beyond the one proposed by the cross-validation or other optimization criterion will not lead to new microstates that survive the statistical tests. 

The microstate segmentation using the cluster analysis can also be applied to spontaneous EEG. It leads to a reduction of the data to a stream of microstates of certain durations, on average around 80–100 msec [33]. It is important to note that in the spontaneous EEG polarity inversion caused by the intrinsic oscillatory activity of the generator processes is ignored. Numerous studies in healthy subjects as well as in patients with different pathologies have shown that a very limited number of map topographies are needed to explain extended periods of spontaneous EEG, and that these few configurations follow each other according to certain rules [34]. We have shown that these different microstates are correlated with well-known fMRI resting states [35]. Analysis of the temporal structure of the microstate transitions showed that the microstates have fractal properties, that is, that their temporal structure is scale invariant over a large time scale [36].

## 5. Statistical Analysis Using CARTOOL

**Figure 5:**
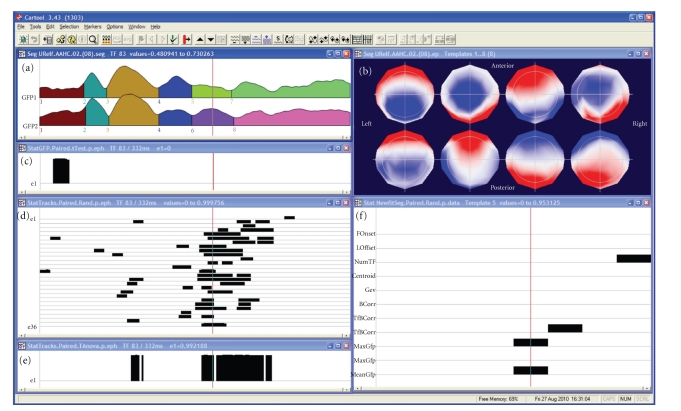
Illustration of the statistical analysis in CARTOOL. The same data as in Figure 2 are used and the segmentation result and the maps of the eight microstates (labeled consecutively) are again shown in the window on the top. The second window on the left shows the test of the Global Field Power (GFP). Black bars indicate time points with *P* < .05. The third window on the left shows the *t*-test for each electrode and each time point. The bottom window shows the test of topographic differences using the TANOVA method. Finally, the window on the right shows the different parameters from the map fitting methods, showing which segments have significant differences in subjects. Note that in this case the topographic analysis corresponds to the two time periods that were significant in the complex electrode-wise *t*-tests, while the GFP test reveals an early effect that was not significant at the single electrode level.

CARTOOL offers a variety of parametric and non-parametric statistical EEG mapping analysis procedures (Figure 5). Non-parametric tests are based on Monte-Carlo bootstrapping methods, while the parametric tests use paired or non-paired *t*-tests. At present, only univariate statistics are implemented in CARTOOL, but multivariate analysis procedures are currently under evaluation before formal inclusion. The univariate analysis can be applied to the potential at each electrode and each time point as a comprehensive exploratory analysis of the data [10, 37, 38]. It is important to note, however, that this analysis is reference-dependent and does not tell us whether topographic or amplitude differences underlie the observed effects (Figure 1). Also the problem for corrections of multiple testing is ill posed. CARTOOL offers Bonferroni corrections as well as the application of the restriction that effects last a certain minimal duration [39]. In order to separately assess strength and topographic differences, CARTOOL proposes statistical analyses using the global variables described above, that is, the GFP and the GMD.

Testing for differences in GFP at each time point is straightforward using the parametric or nonparametric tests. In order to test for differences in topography, CARTOOL implemented what has been called a “topographic ANOVA”, or TANOVA [17, 40]. Since GMD is a single measure of difference between the maps of two conditions, mean and standard error of topography for each condition/population cannot be calculated. The way to overcome this problem is to perform a non-parametric randomization test based on the GMD values. This is done in the following way: (1) assigning the maps of the single subject in a randomized fashion to different experimental conditions, (2) recalculating the group-average ERPs, and (3) recalculating the resulting GMD value for these “new” group-average ERPs. The number of permutations that can be made with a group-average ERP based on *n* participants is 2^*n*^. The GMD value from the actual group-average ERPs is then compared with the values from the empirical distribution to determine the likelihood that the empirical distribution has a value higher than the GMD from the actual group-average ERPs. In a within-subject design, the permutation of the maps is done within the subjects, while the permutation is done across subjects in group comparisons. In the example shown in Figure 5, the TANOVA is performed between two conditions for each time point. The figure illustrates the simplicity of the analysis of the global parameters GFP and GMD compared to the electrode-wise statistics (besides the fact that the latter are reference independent). However, it is important to note here that a latency shift of one condition with respect to the other could lead to strong and long topographic differences that are in fact not due to different areas being activated by the two conditions, but by activations of the same areas at different moments in time. The comparison of the TANOVA result with the microstate segmentation is therefore important.

The statistical analysis can also be performed on the values that result from the microstate fitting procedure. Again, only univariate statistics are currently implemented. For multivariate analysis the spreadsheets have to be read into other statistical packages. Ongoing developments are underway for the implementation of multivariate statistics [41, 42] as well as other topographic analysis methods such as the test for ERP component stability recently proposed by Koenig and Melie-García [16].

## 6. Frequency Analysis of Multichannel EEG

Quantitative analysis of spontaneous EEG has traditionally relied on Fourier-transformation based spectral analysis. Thereby, the power of the different frequencies or frequency bands is compared between different conditions/populations. In multichannel data, power maps are often used and statistical maps of power differences are calculated. This approach has been very successful and helped to characterize vigilance changes, sleep stages, drug effects and various neurological and psychiatric disorders [43]. More recently, time resolved frequency analysis has been applied to spontaneous EEG using wavelet procedures [44]. CARTOOL has implemented these frequency analysis methods using FFT as well the S-transform. Windows can flexibly be defined with variable amount of overlap.

However, frequency power maps have two important problems. First, they are reference-dependent. In contrast to potential maps in the time domain, power maps in the frequency domain change when the position of the recording reference changes [45, 46]. Second, power maps ignore the phase differences between the electrodes. Only the amplitude is considered. Therefore, source localization of power maps is not possible [47]. Ignoring the phase relationship between electrodes is unfortunate, because they are determined by the configuration and interaction of the intercerebral sources. In order to perform source localization in the frequency domain, the inverse solutions have to be calculated for the complex data derived from the FFT. Consequently, inverse solutions in the frequency domain are initially complex [48]. An efficient way to perform distributed source localizations of a large number of EEG epochs in the frequency domain is to first compute average cross-spectral matrices [49]. Another, more simplified way is to approximate the power maps by maps where all electrodes have a common phase. This method has been called FFT-approximation [47]. It is based on the calculation of the first principal component of the data in the complex plane. The results are single-phase approximated potential map for each frequency that can be subjected to source estimation methods. It has initially been developed for single equivalent dipole localization methods, because a single dipole cannot account for phase differences between electrodes. However, distributed source estimation algorithms can also be applied to these maps in order to localize the distribution of all sources that are oscillating in phase. The FFT-Approximation method is implemented in CARTOOL.

## 7. Source Localization

While the analysis of the scalp potential maps as described up to now has the advantage, as compared to waveform analysis, to be reference independent and considers the whole brain electrical activity, it does not provide any direct conclusions about the number, location and orientation of the intracranial generators [50]. Inverse solution methods are required to estimate these sources.

**Figure 6:**
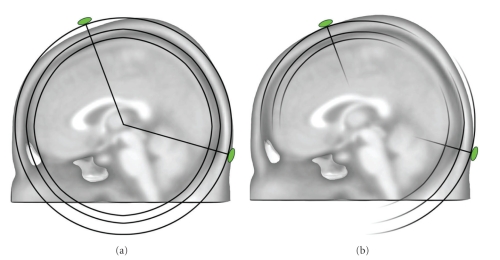
Illustration of the two head models used for the inverse solution calculation in CARTOOL. The SMAC model (a) uses 3-shell of constant radiuses for the scalp and skull, which is in average a good approximation, but can be locally inaccurate for some electrodes. Due to the spherization step, the geometrical relationship between the inverse space and the electrodes is also slightly incorrect. The LSMAC model (b) uses the local radiuses of the scalp and skull, under each electrode locus, to generate different sets of 3 shells spherical model. Therefore, the forward problem is geometrically correct for each electrode.

A major breakthrough in the spatial analysis of multichannel EEG/MEG was the development of distributed inverse solution methods that allow the estimation of the 3-dimensional distribution of neuronal activity in the whole brain at each moment in time [3, 51, 52]. The stability and reliability of these methods are impressive, and they have been validated by several direct comparisons with intracranial recordings, lesion studies and other neuroimaging methods [53]. The advancement in this field has tremendously boosted the use of electrophysiological methods in experimental and clinical studies because of the major advantages that the high temporal resolution provides. CARTOOL has implemented some of the major distributed linear inverse solutions, namely the weighted minimum norm solution (WMN) [54], the low resolution electromagnetic tomography (LORETA) [55] and the local autoregressive average (LAURA) [56] and EPIFOCUS [57]. CARTOOL calculates the inverse matrices for these different source models. It is well known that the regularization parameter can strongly influence the inverse solutions. It cannot only eliminate, but also create “ghost sources” in case of overfitting the data [58]. CARTOOL uses the L-curve method [59] to find the optimal regularization value for a given data file. This optimal value is used as default display, but the user has the possibility to toggle through stronger and weaker regularizations that are also stored in the inverse matrix. The inverse matrices are multiplied online with the EEG data and displayed for each time point using different display options. Currently, the so-called SMAC (Spherical Model with Anatomical Constraints) head model [60] as well as a more complex head models based on local spheres are available, both applied either to the individual MRI (if available) or to template MRIs. In the SMAC model, the full head is transformed to a sphere through a non-linear warping function based on the surface of the scalp. The mean radius of the scalp, skull and brain are then used for a 3-shell model. Depending on brain size, between 3000 and 5000 solution points are then defined in regular distances within the gray matter. This also includes deeper brain structures such as the amygdala, hippocampus and thalamic structures, as long as they are recognized as grey matter. The forward problem is then solved with an analytical solution using this “realistic” (i.e., individual) head model without any constraints on dipole orientation. The LSMAC model (Locally Spherical Model with Anatomical Constrains), on the contrary, does not need this initial spherization step. Instead, at each electrode locus, an adaptive local spherical model is used. To do so, and sequentially under each electrode, the thicknesses of the scalp, skull and brain are estimated. These thicknesses are then used in a 3-shell spherical model with the local radiuses, allowing the real geometry between solution points and electrodes to be accounted for. The SMAC and LSMAC methods are illustrated in Figure 6.

**Figure 7:**
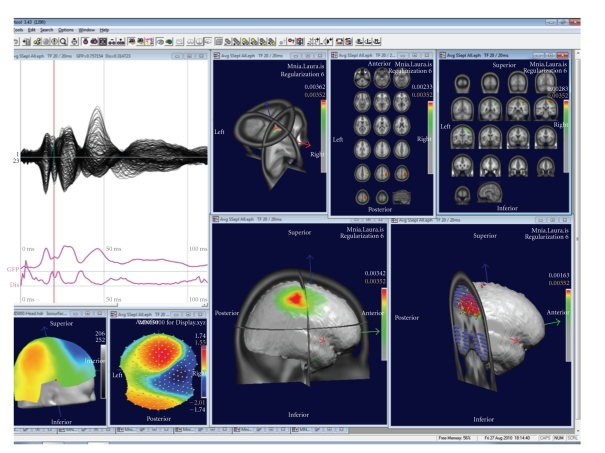
Examples of displays of the source localization results in CARTOOL. The data show a grand-mean somatosensory evoked potential after left median nerve stimulation. Overlapped waveforms and the map at 20 ms are displayed on the left. The right windows show different types of displays of the sources at this time point.

The results of the inverse solutions (norm or vectors) are displayed 3-dimensionally in the real (i.e., untransformed) MRI (Figure 7). Slices in all orientations can be shown as well as the solutions on surface-rendered images. The inverse solution results can be stored as matrices for further statistical processing (source waveform analysis), or as volumes for fusioning with other neuroimaging results or for importing into other image analysis tools such as SPM.

It is worthwhile to note that the segmentation of the brain surface and the grey matter is implemented in CARTOOL including manual correction tools to exclude incorrect classification of grey matter or exclude structures as brainstem and cerebellum if desired. Alternatively, already segmented brains can be read into CARTOOL if preferred, such as standard template brains. Also the warping transformations as well as the distribution of the solution points are done within CARTOOL. Thus, the whole source localization process can be performed within CARTOOL, starting with the original EEG and the original MRI.

Besides source reconstruction in the individual MRI, CARTOOL can also use template brains such as the MNI brain. In this case the solution space remains the same for all subjects, allowing group studies on the source level. In the case of the MNI brain, all solution points are labeled with their Talairach coordinates as well as their anatomical labels. Time periods of interest (e.g., the microstates) or regions of interest (ROI) can be created and the mean current density within these segments or ROI can be stored for further statistical analysis [61]. Finally, the source waveforms can be stored and further treated in CARTOOL like scalp electrode traces.

## 8. Additional Implementations

Several other analysis tools are available in CARTOOL. In addition to the analysis of scalp EEG, CARTOOL also allows to visualize and analyze intracranial EEG, to read and fusion 3D images of different imaging modalities, to create and work with 3D regions of interest, and to convert and manipulate MR images.

**Figure 8:**
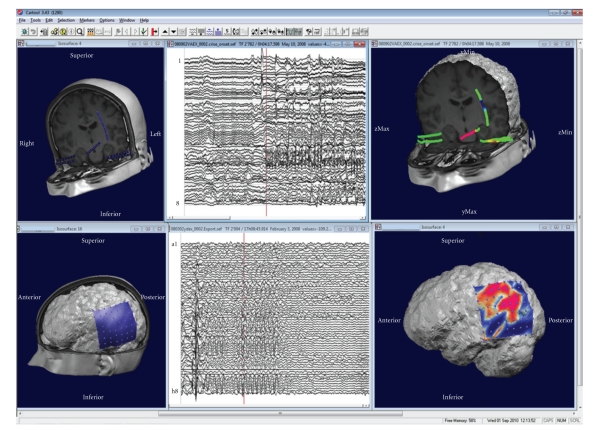
Illustration of the display of intracranial recordings using CARTOOL. Top: recordings from depth electrodes: Left: display of the electrode positions. Middle: waveform display of the beginning of a seizure. Right: potential mapped on the electrodes. Bottom: recordings from a 8 × 8 subdural grid. Left: display of the grid position on the patient's MRI. Middle: Traces from the 64 electrodes. Right: potential mapped on the grid for one moment in time.

The analysis of intracranial recordings includes the color-coded mapping of grids as well as along depth electrodes (Figure 8). Intracranial electrodes are directly fused with the patient's MRI if the exact positions are available. Depth shifting tricks allow the experimenter to see the potentials of the depth electrodes within the MRI. 

CARTOOL can read any type of 3D volumes in Analyze format. In case of functional images (fMRI or PET) the activation areas are displayed as colored blobs within the MRI. Several different volumes can be overlapped and thus results of different imaging modalities (including the inverse solutions) can be displayed within the same MRI. MRIs can also be manipulated and converted in various ways and stored as new volumes.

**Figure 9:**
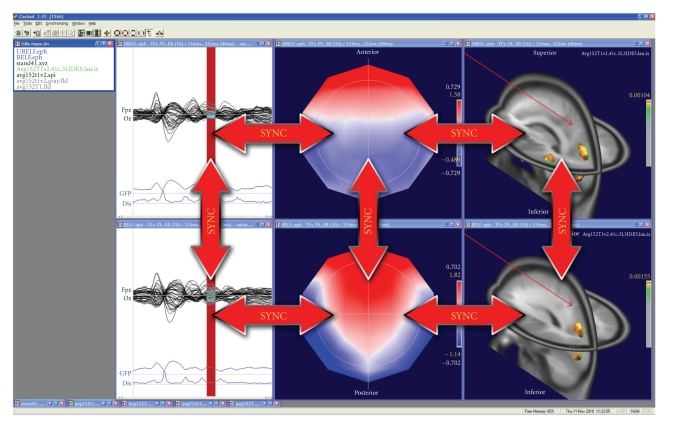
Illustration of the synchronization of windows in CARTOOL. Synchronization within the same dataset allows dynamic animations in time. Synchronization across datasets allows comparison of different conditions in time and in space.

A large palette of 3D display tools is available in CARTOOL that allows flexible visualization of the data. Any 3D object can be inserted into another one by a single click, allowing the merging of different information instantly, while retaining the spatial coherence of the objects. Different windows can easily be synchronized, allowing the user to visualize the dynamic behavior of the data on traces, maps and inverse solutions simultaneously (Figure 9).

## 9. Ongoing Developments

CARTOOL is constantly implementing new analysis methods that appear as useful and have been published. Concerning the statistical analysis, multivariate methods will soon be implemented. The TANOVA described above can easily be extended to multivariate measures [42]. Also tests of topographic consistency across subjects based on randomization tests of GFP are promising and will be implemented soon [16].

Concerning microstate segmentation, De Lucia et al. [31, 62] proposed a variation of the cluster analysis described above to apply to single-trial ERP data. The method proposes to model the overall electrical response, that is, the event-related and the ongoing activity, as a mixture of Gaussians, in an *N*-dimensional space, where *N* is the number of the electrodes. The computation is initialized by a K-means algorithm, which iteratively improves the estimation of means, covariances and priors of the Q Gaussians until the likelihood reaches a plateau. For each time point and trial it then provides Q conditional probabilities that relate the topographies to the clusters. Like in the labeling procedure described above, each time point and trial is then labeled with the cluster with which it has highest conditional probability. The method has been shown to reliably identify specific component maps in the single trial ERPs despite the clear dominance of the ongoing spontaneous EEG activity.

An important new development in the field of EEG/MEG analysis is the measure of connectivity between different brain areas as a way to understand the organized behavior of different brain regions [63]. The use of EEG data to examine the functional connectivity has a long history [64, 65] and a variety of techniques have been used, most prominently the calculation of cross-correlation or phase synchronization between pairs of scalp electrodes or sensors [66]. Also graph-theory-based tools from the study of complex network have been proposed [67]. The problem with such analysis on the scalp surface is that the interpretation with respect to the sources that generated the connectivity between electrodes is ambiguous because of the spreading of electromagnetic signals from the cortex to the sensors. More appropriate is the use of connectivity measures in the inverse space. A popular method that is often applied to MEG data (but can also be used for EEG) is the so-called dynamic imaging of coherent sources (DICS), proposed by [68]. This method uses a beamformer spatial filter to identify coherent sources in the brain for specific frequency bands. Like other spectral coherence methods, DICS does not give information of the direction of information flow. Several alternative methods have therefore been proposed that are based on the Granger causality theory and multivariate autoregressive models such as Partial Directed Coherence [69] or the Directed Transfer Function [70]. Applying these methods to the data in the inverse space after applying the distributed linear inverse solutions described above will allow estimating the flow of electrical information in large-scale neuronal networks in real time. Such methods will be implemented in future versions of CARTOOL together with other promising distributed inverse solutions and head models that currently emerge in the literature.

## 10. Software Details

CARTOOL is not an Open Source project; however the program is distributed freely to any nonprofit research group. Users need to register only once and will then be informed of any updates of the software. They are asked to cite the use of CARTOOL in the *Methods* and *Acknowledgements* sections of their papers. Currently about 650 users from all over the world have registered and downloaded CARTOOL.

CARTOOL runs only on Windows platforms (ranging from Windows 95 up to Windows 7) as a standalone compiled executable program, included with its complete documentation within a single installation program. It is fully written in C++ in order to attain the highest speed and compactness in memory use. The advanced display is completely done in *OpenGL*, so it needs an *OpenGL* accelerated graphic card, and as much memory as possible for the most demanding operations. Matlab is not needed to run CARTOOL.

Interoperability is achieved mainly by exchanging intermediate results through files. Considerable efforts have been put in reading and writing standard files with a maximum of convenience for the user. For example, most of the operations can be done by *Drag & Drop* in CARTOOL, the landing of the drop usually conditioning the actions to be undertaken on the files.

Help is provided at different levels. A thorough *Reference Guide*, describing all the options and technical details for all processes, is included in the distribution in the form of compiled HTML (10 MB *chm* file). In addition a CARTOOL* Community* group (Google Sites) has been built in order to offer a central access for the users, which includes a *User's Guide*, collaboratively edited as a Wiki, a *Discussion Forum* for all questions and announcements, some *FAQs*, and a few shared files. Finally, it is worth noting that CARTOOL updates, including Beta releases, can be easily assessed online through a Google Docs repository.

Here are the main internet addresses for CARTOOL:

http://brainmapping.unige.ch/cartool
http://cartoolcommunity.unige.ch

Files formats read by CARTOOL include formats produced by the EEG systems form the companies Biologic, Biosemi, Brain Products, Deltamed, EGI, and Neuroscan. Also EDF format and standard text files can be read. Concerning MRI, Analyze and AVS formats are read. A complete list of file formats is included in the Reference Guide of Cartrool.
